# Development and Validation of a Prediction Model for Anxiety Improvement after Deep Brain Stimulation for Parkinson Disease

**DOI:** 10.3390/brainsci13020219

**Published:** 2023-01-28

**Authors:** Bowen Chang, Jiaming Mei, Chen Ni, Chi Xiong, Peng Chen, Manli Jiang, Chaoshi Niu

**Affiliations:** 1 Department of Neurosurgery, The First Affiliated Hospital of USTC, Division of Life Sciences and Medicine, University of Science and Technology of China, Hefei 230001, China; 2 Anhui Province Key Laboratory of Brain Function and Brain Disease, Hefei 230001, China

**Keywords:** deep brain stimulation, Parkinson’s disease, anxiety, non-motor symptoms, nomogram

## Abstract

Background: Parkinson’s disease (PD) represents one of the most frequently seen neurodegenerative disorders, while anxiety accounts for its non-motor symptom (NMS), and it has greatly affected the life quality of PD cases. Bilateral subthalamic nucleus deep brain stimulation (STN-DBS) can effectively treat PD. This study aimed to develop a clinical prediction model for the anxiety improvement rate achieved in PD patients receiving STN-DBS. Methods: The present work retrospectively enrolled 103 PD cases undergoing STN-DBS. Patients were followed up for 1 year after surgery to analyze the improvement in HAMA scores. Univariate and multivariate logistic regression were conducted to select factors affecting the Hamilton Anxiety Scale (HAMA) improvement. A nomogram was established to predict the likelihood of achieving anxiety improvement. Receiver operating characteristic (ROC) curve analysis, decision curve analysis (DCA), and calibration curve analysis were conducted to verify nomogram performance. Results: The mean improvement in HAMA score was 23.9% in 103 patients; among them, 68.9% had improved anxiety, 25.2% had worsened (Preop) anxiety, and 5.8% had no significant change in anxiety. Education years, UPDRS-III preoperative score, and HAMA preoperative score were independent risk factors for anxiety improvement. The nomogram-predicted values were consistent with real probabilities. Conclusions: Collectively, a nomogram is built in the present work for predicting anxiety improvement probability in PD patients 1 year after STN-DBS. The model is valuable for determining expected anxiety improvement in PD patients undergoing STN-DBS.

## 1. Introduction

Parkinson’s disease (PD) represents one of the progressively degenerative neurological diseases that usually occurs among the elderly, with a higher incidence rate in males than in females. For PD cases, resting tremors, rigidity and bradykinesia are the major motor symptoms [1]. Moreover, various non-motor symptoms (NMS) can also be observed in such cases, such as mood disorders, cognitive impairment, sleep disturbances, and autonomic dysfunction [2,3]. Typically, concurrent mood disorders, such as bipolar disorder, anxiety and depression, have a negative influence on the clinical course and life quality of OD cases. Anxiety, one of the frequent NMS among PD cases, is characterized by increased feelings of fear, apprehension and worries [4,5]. With the development of neuromodulation therapy in recent years, deep brain stimulation (DBS) has emerged as an efficient way to treat advanced PD [6,7]. DBS has been extensively suggested previously to remarkably enhance motor symptoms among PD cases; however, it is still controversial whether DBS is effective in improving anxiety in these patients [8,9]. Similarly, clinical models predicting depressed mood improvement among PD cases receiving DBS are lacking. Effective models have been developed by integrating several important factors and building models for predicting the probability of anxiety improvement among PD cases receiving DBS surgery. The present work retrospectively enrolled PD cases receiving bilateral subthalamic nucleus DBS (STN-DBS) at our institute to develop and validate an effective model for predicting anxiety improvement among PD cases following STN-DBS.

## 2. Materials and Methods

### 2.1. Patients

Between September 2019 and April 2021, questionnaire results and medical records for PD cases receiving STN-DBS were extracted at First Hospital of the University of Science and Technology of China. Our study protocols gained approval from the Ethics Committee of our institute (2022-RE-154). Patients with intermediate-to-advanced PD were enrolled. Meanwhile, those with persistent severe psychiatric disorders, severely impaired cognition, diffusion ischemia or severe atrophy observed from brain MRI, and those with systemic concurrent illnesses preventing surgical treatment were excluded from this study. 

### 2.2. Outcome Assessment

This study obtained clinical information from questionnaires and medical records of eligible cases, which included age, sex, levodopa equivalent dose (LED) and course of disease. Symptom severity, psychological status, cognitive status, and life quality of patients were assessed by detailed scales. Symptom severity was evaluated by the UPDRS-III scale, whereas the NMS of PD was evaluated by the Parkinson’s Disease Non-motor Symptom Scale (NMSS), and life quality was evaluated by the PDQ-39 scale. In addition, the psychological status of patients was evaluated by Hamilton Anxiety (HAMA) together with Hamilton Depression (HAMD) scales, and their cognitive status was evaluated by the Mini-Mental State Examination (MMSE) and the Montreal Cognitive Assessment (MoCA) scales. After 1 year, the MoCA scale was reused to assess the cognitive status of patients. PD cases were classified as anxiety-improved or non-improved groups based on whether their HAMA scores improved 1 year after surgery.

### 2.3. Statistical Analysis

Statistical analysis was performed using R (http://www.R-project.org, X&Y solutions, Inc. Boston, MA, USA, accessed on 1 June 2022) and Empower(R) (www.empowerstats.com, X&Y solutions, Inc. Boston, MA, USA, accessed on 1 June 2022 ). First, the Kolmogorov–Smirnov test was conducted to test whether the variables were normally distributed. Second, normally distributed variables were assessed by one-way ANOVA or a two-tailed Student’s *t*-test. Non-parametric variables were compared by the Mann–Whitney U test across diverse groups simultaneously. Risk factors were explored using multivariate logistic regression. Third, a regression model was constructed based on the identified risk factors. The model was later converted to a nomogram. Afterwards, receiver operating characteristic (ROC) curve, decision curve analysis (DCA) and calibration curve analysis were performed to assess model performance. Pearson’s correlation coefficient test was then performed to analyze the associations between improvement in HAMA score and education, HAMA preop, UPDRS-III drug-on, and UPDRS-III drug-off scores. 

## 3. Results

### 3.1. Patients

**Figure 1:**
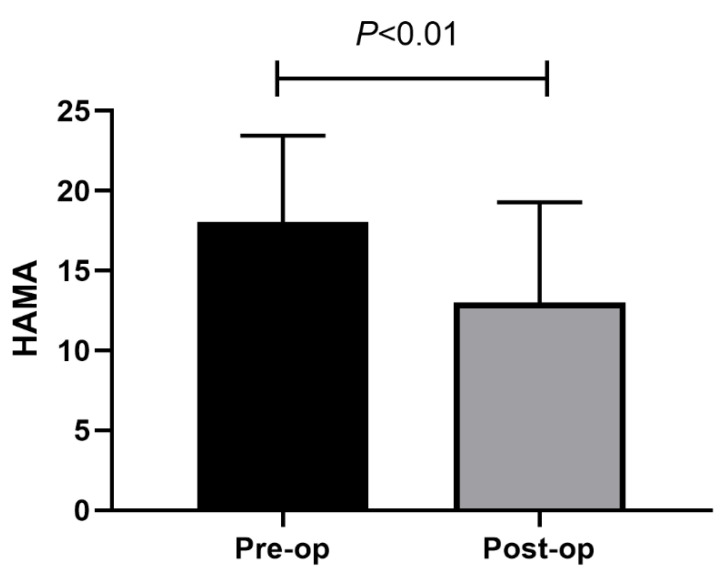
Comparison of HAMA score between pre-operation and 1 year after the operation.

A total of 103 PD cases were enrolled in this study. Table 1 shows the demographic data of the enrolled PD cases. There were 63 males (61.17%) and 40 females (38.83%) with PD cases, with an age of 35–75 years. The average and median HAMA score improvement rates were 23.9% and 25.4%, respectively, at 1-year post-STN-DBS. Relative to those before surgery, STN-DBS remarkably increased HAMA scores in PD cases (Figure 1). Furthermore, 68.9% of the patients’ anxiety improved, 25.2% worsened, and 5.8% did not exhibit any significant change. All cases were classified into two groups according to whether their anxiety had improved or not.

**Figure 2:**
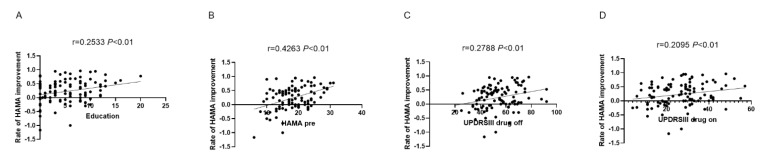
Correlations of HAMA score improvement rate with education (**A**), HAMA preop (**B**), UPDRSIII drug off (**C**) and on (**D**).

The improvement group included 42 males (59.15%) and 29 females (40.85%), and the average age was 58.58 years. In contrast, the non-improvement group included 21 males (65.62%) and 11 females (34.38%), and the average age was 59.97 years. Education years, HAMA Preop, PDQ-39 Preop, UPDRS-III drug-on and UPDRS-III drug-off scores were not significantly different in both groups. Table 2 displays the univariate analysis results for all variables. The anxiety improvement was related to education years, HAMA Preop, PDQ-39 Preop, UPDRS-III drug-on and UPDRS-III drug-off scores. Table 3 shows the multivariate regression results for all variables between the higher and lower improvement rate groups. Education years, HAMA Preop, UPDRS-III drug-on, and UPDRS-III drug-off independently predicted HAMA score improvement among PD cases receiving STN-DBS. Additionally, education years, HAMA Preop, PDQ-39 Preop, UPDRS-III drug-on and UPDRS-III drug-off scores in PD cases showed a positive relation to HAMA score improvement at 1-year postoperatively (Figure 2).

### 3.2. Development of the Nomogram

**Figure 3:**
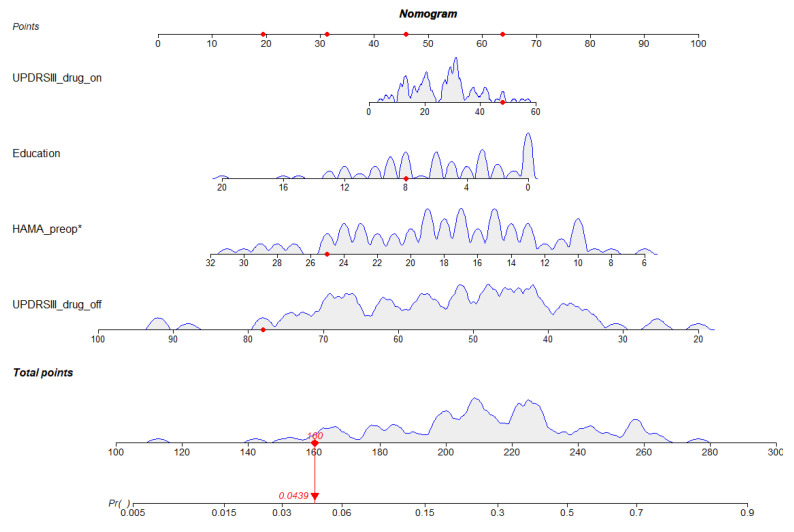
Nomogram to predict improvement of HAMA score after STN-DBS for PD. The clinical factor corresponds to a specific point by drawing a line straight upward to the point’s axis. After the sum of the points is located on the total points axis, the sum represents the probability of getting an HAMA score improvement. *, *p* < 0.001.

**Figure 4:**
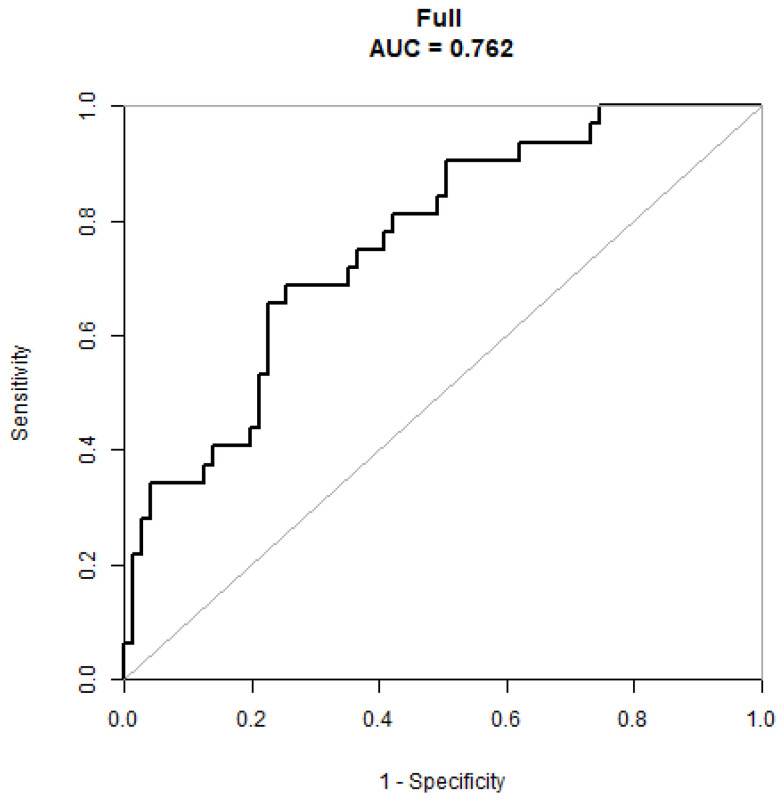
A receiver operating characteristic curve to evaluate the discriminating capability of the nomogram.

According to the findings above, this work developed the prediction model and generated a column line plot for predicting HAMA score improvement at 1-year post-STN-DBS treatment (Figure 3). Clinical factors indicated diverse scores, while the linear point axis was utilized to calculate the total score, which represented the increased likelihood of improvement in HAMA scores. Figure 4 shows that our prediction model performed well in discrimination. The area under the ROC curve (AUC) value was 0.76, and the C-index value was 0.76.

### 3.3. Validation of the Nomogram

**Figure 5:**
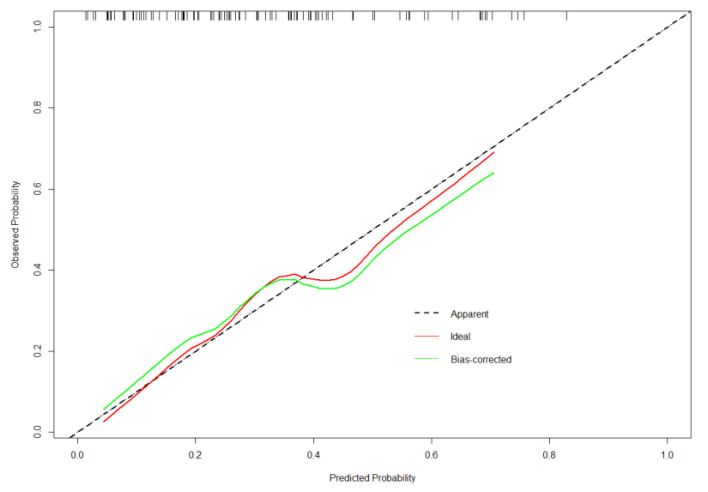
Calibration curve of the model. The calibration of the model is in line with the agreement between predicted and observed outcomes of improvement of the HAMA score.

**Figure 6:**
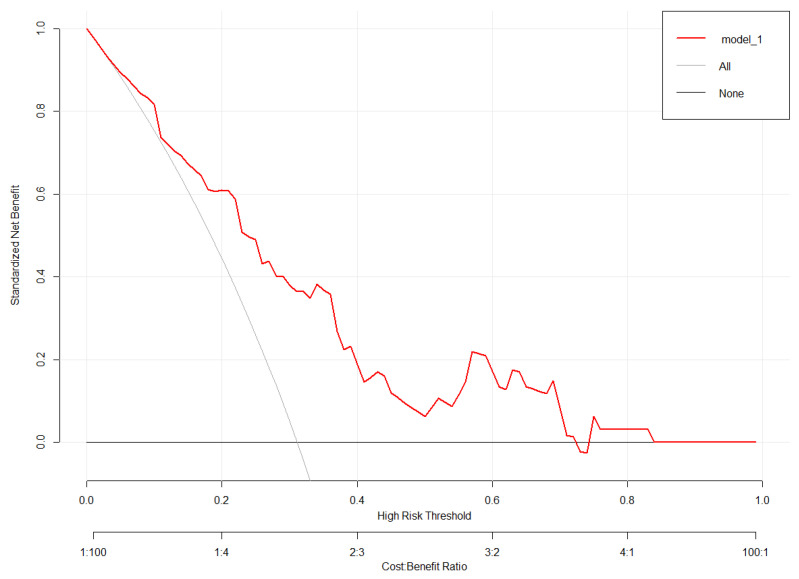
The decision curve analysis diagram of the model.

A bootstrap validation approach was utilized for the internal validation of our model, and the bootstrap-corrected C-index was 0.72. In addition, the model-predicted and real improvements were plotted to obtain the calibration curves between H-score improvement (X-axis) and HAMA score improvement (Y-axis). As shown in Figure 5, our predicted improvement was consistent with the observed improvement. In addition, DCA was conducted, where the net benefit rate was utilized to be ordinal, whereas the high-risk threshold was set to be negative (0.1) (Figure 6). According to Figure 4, the net benefit rate was greater than 0 at the high-risk threshold of 0–1, which was clinically significant.

## 4. Discussion

Anxiety is a common psychological disorder associated with PD. The incidence of anxiety disorders is high among PD cases, which is possible because of the heterogeneities in anxiety measurement instruments, current anxiety definitions and study sample features [10,11]. One cross-sectional study using ICD-9.22 to diagnose psychiatric disorders reported an incidence as low as 12.8%. Moreover, the incidence of current anxiety can be as high as 43% in PD cases, as measured by the DSM-IV criteria. DBS is suggested as effective in treating intermediate-to-advanced PD cases, which can improve motor symptoms, disability and fluctuations, along with patient life quality. In recent studies, DBS has been found to be effective in PD cases showing early motor complications, which suggests that DBS can be applied in more people than before [12,13,14]. Nonetheless, DBS may be associated with psychiatric and cognitive side effects among PD cases. As reported in some studies, patient outcomes may differ among the different target sites chosen, i.e., the globus pallidus internus (GPi) or subthalamic nucleus (STN) [15,16]. Besides, it is also suggested that DBS does not lead to obvious side psychiatric effects on PD cases [17,18]. According to one resting-state functional MRI-based research, anxious PD patients had reduced connectivity in the sensorimotor network (SMN) and default mode, whereas they had enhanced connectivity in the executive control network (ECN) compared with non-anxious PD patients [19]. Moreover, since STN-DBS modulates sensory networks and is beneficial for motor deficits, stimulation of the anterior and limbic STN may also improve troublesome behavioral deficits, which may account for the improved anxiety symptoms among PD cases receiving STN-DBS, but this requires further functional imaging studies [20]. The present work enrolled altogether 103 PD cases for analyzing factors associated with their ability to achieve improvement in anxiety within 1 year after STN-DBS. According to our results, STN-DBS was effective in improving patients’ anxiety. At 1 year after surgery, 68.9% of patients had improved anxiety, 25.2% had worsened cognitive status, and 5.8% had no change in cognitive status. Based on the above results, exploring the factors affecting the benefits of improving anxiety status among PD cases receiving STN-DBS is of great importance.

Overall, DBS can improve anxiety in PD cases. It is possibly associated with reduced motor symptoms alone and a declined dependence on family and friends for support [21]. As reported in another meta-analysis, age, stimulation course and LED during the follow-up period are related to aggravated anxiety symptoms after surgery [22]. This study found that improvement in patient anxiety 1 year postoperatively was possibly associated with the education years, HAMA Preop, UPDRS-III drug-on Preop, and UPDRS-III drug-off Preop scores of patients. The present work further analyzed the above factors. As confirmed by multivariate regression, the above-mentioned clinical factors were the independent risk factors for the improvement of anxiety 1 year after surgery. It is well-known that access to education can enhance the mental health of human beings. In one study, an additional year of education was found to result in a lower likelihood of reporting depression- and anxiety-related symptoms. People who receive high education levels may not experience severe depression and anxiety symptoms [23]. According to our results, cases with high education levels showed better improvement in depression 1 year after surgery. In addition, the improvement rate of HAMA scores was positively associated with HAMA Preop, and it independently predicted an improvement in anxiety obtained by patients. Cases developing severe anxiety before surgery may be more likely to have an improvement in anxiety, thus increasing the improvement rate. Consequently, patients’ anxiety levels should be fully assessed preoperatively so as to reasonably expect anxiety improvement post-treatment. Additionally, UPDRSIII Preop showed a positive relation to the HAMA score improvement rate, and it independently predicted postoperative anxiety improvement. According to the above analyses, severe motor symptoms before surgery may induce a superior improvement in anxiety after surgery. 

Given its clinical heterogeneity, the improvement in anxiety after STN-DBS may be multifactorial and associated with improved motor function, sleep quality, quality of life and reduced medication in individual patients. However, the interactive test showed that the independent factors had a stable effect on the improvement rate of anxiety in both the low motor symptom improvement rate and the improvement rate groups.

However, the likelihood of improvement in anxiety is quite different, even when the cases display identical risk factors. Improvements in anxiety may result from several factors. Univariate regression possibly has a unilateral effect on prognosis, which usually ignores additional important factors and thereby makes it impossible to accurately judge the outcomes of patients. A nomogram is a clinical prognostic prediction tool that combines the influences of different factors on outcomes of patients. It has been extensively adopted to analyze cancer patient survival and replaces conventional prediction models. This study included 103 patients in the model and constructed the initial nomogram model to predict improvements in cognitive status among PD cases receiving STN-DBS. Education years, UPDRS-III drug-on Preop, UPDRS-III drug-off Preop, and HAMA Preop were used as the nomogram scores through multifactorial analysis. Our constructed model achieved favorable prediction ability, with a C-index value of 0.76, consistent with the calibration plot analysis based on our validation cohort.

Finally, certain limitations should be noted in the present work. First, the present work was a single-center study. It is unclear whether factors such as diet, race and climate have an impact on the probability of improvement in the anxiety of PD cases. In addition, the feasibility of our model in primary care or additional fields remains to be confirmed. Our next step will be to use a multicenter collaborative approach with randomly selected PD patients at additional centers for external validation. Second, bias in follow-up cannot be avoided due to the retrospective nature of this work. 

## 5. Conclusions

Collectively, a nomogram is built in the present work for predicting the probability of improvement in anxiety among PD cases receiving 1 year following STN-DBS. The model is valuable for determining expected anxiety improvement in PD patients undergoing STN-DBS.

## Figures and Tables

**Table 1:** Comparison between patients with and without improved anxiety after STN-DBS.

	Improvement Group	Non-Improvement Group	*p*-Value
No.	71	32	
Age (years)	58.58 ± 8.24	59.97 ± 7.57	0.418
Education (years)	6.18 ± 4.56	4.11 ± 3.62	0.025
LED	658.10 ± 409.99	664.06 ± 236.40	0.939
Drug improvement rate	0.51 ± 0.15	0.54 ± 0.13	0.304
UPDRSIII drug off	56.86 ± 13.72	47.66 ± 13.16	0.002
UPDRSIII drug on	28.28 ± 11.57	23.00 ± 10.51	0.030
NMSS Preop	90.32 ± 28.71	81.34 ± 32.31	0.161
PDQ39 Preop	75.42 ± 16.52	68.41 ± 14.74	0.042
MOCA Preop	17.92 ± 5.89	20.34 ± 6.49	0.064
MMSE Preop	24.89 ± 3.54	25.22 ± 4.20	0.679
HAMD Preop	16.58 ± 7.06	13.84 ± 5.31	0.054
HAMA Preop	19.20 ± 5.27	15.53 ± 4.81	0.001
Duration (years)	8.90 ± 3.88	8.25 ± 3.63	0.423
Gender			0.533
male	42 (59.15%)	21 (65.62%)	
female	29 (40.85%)	11 (34.38%)	
H-Y			0.537
2	2 (2.82%)	0 (0.00%)	
2.5	9 (12.68%)	7 (21.88%)	
3	34 (47.89%)	16 (50.00%)	
4	20 (28.17%)	8 (25.00%)	
5	6 (8.45%)	1 (3.12%)	

**Table 2:** Effect of characteristics of patients on the improvement of anxiety.

	Statistics	OR (95% CI) *p*-Value
Age (years)	59.01 ± 8.03	1.02 (0.97, 1.08) 0.4146
Education (years)	5.54 ± 4.38	0.89 (0.80, 0.99) 0.0287
LED	659.95 ± 363.80	1.00 (1.00, 1.00) 0.9383
Drug improvement rate	0.52 ± 0.14	4.78 (0.25, 92.97) 0.3019
UPDRSIII drug off	54.00 ± 14.15	0.95 (0.91, 0.98) 0.0034
UPDRSIII drug on	26.64 ± 11.47	0.96 (0.92, 1.00) 0.0332
NMSS Preop	87.53 ± 30.01	0.99 (0.98, 1.00) 0.1623
PDQ39 Preop	73.24 ± 16.25	0.97 (0.95, 1.00) 0.0469
MOCA Preop	18.67 ± 6.15	1.07 (1.00, 1.15) 0.0661
MMSE Preop	24.99 ± 3.74	1.02 (0.91, 1.15) 0.6758
HAMD Preop	15.73 ± 6.66	0.93 (0.87, 1.00) 0.0565
HAMA Preop	18.06 ± 5.38	0.86 (0.79, 0.95) 0.0022
Duration (years)	8.70 ± 3.80	0.95 (0.85, 1.07) 0.4201
Gender		
male	63 (61.17%)	1.0
female	40 (38.83%)	0.76 (0.32, 1.81) 0.5335
H-Y		
2	2 (1.94%)	1.0
2.5	16 (15.53%)	4,478,298.90 (0.00, Inf) 0.9881
3	50 (48.54%)	2,709,559.00 (0.00, Inf) 0.9885
4	28 (27.18%)	2,303,125.15 (0.00, Inf) 0.9886
5	7 (6.80%)	959,635.48 (0.00, Inf) 0.9893

**Table 3:** Multivariate regression showing the effect of education, UPDRS-III drug on, UPDRS-III drug off, PDQ-39 Preop, and HAMA Preop on the improvement of anxiety.

	Non-Adjusted		Model I		Model II	
	OR (95% CI)	*p*-Value	OR (95% CI)	*p*-Value	OR (95% CI)	*p*-Value
Education (years)	0.89 (0.80, 0.99)	0.0287	0.88 (0.79, 0.99)	0.0266	0.88 (0.79, 0.99)	0.0259
UPDRSIII drug off	0.95 (0.91, 0.98)	0.0034	0.95 (0.91, 0.98)	0.0026	0.95 (0.91, 0.98)	0.0028
UPDRSIII drug on	0.93 (0.87, 0.99)	0.0332	0.95 (0.91, 0.99)	0.0203	0.94 (0.86, 1.02)	0.1110
PDQ-39 Preop	0.97 (0.95, 1.00)	0.0469	0.97 (0.94, 1.00)	0.0282	0.97 (0.94, 1.01)	0.1379
HAMA Preop	0.86 (0.79, 0.95)	0.0022	0.86 (0.78, 0.94)	0.0017	0.86 (0.77, 0.96)	0.0061

Model I is adjusted for age, LED, duration and gender, whereas Model II is adjusted for age, LED, duration, gender, education, H-Y, LED, NMSS Preop, MoCA Preop, MMSE Preop and HAMD Preop. CI, confidence interval; OR, odds ratio.

## Data Availability

The original contributions presented in the study are included in the article/Supplementary Material; further inquiries can be directed to the corresponding author.
